# Diversity, distribution, and drivers of *polychromophilus* infection in Malagasy bats

**DOI:** 10.1186/s12936-021-03696-0

**Published:** 2021-03-20

**Authors:** Mercia Rasoanoro, Steven M. Goodman, Milijaona Randrianarivelojosia, Mbola Rakotondratsimba, Koussay Dellagi, Pablo Tortosa, Beza Ramasindrazana

**Affiliations:** 1 Institut Pasteur de Madagascar, Antananarivo 101, BP 1274, Ambatofotsikely, Madagascar; 2 Faculté des Sciences, Université d’Antananarivo, BP 706, Antananarivo 101, Antananarivo, Madagascar; 3 Association Vahatra, Antananarivo 101, BP 3972, Antananarivo, Madagascar; 4 Field Museum of Natural History, 1400 South Lake Shore Drive, 60605 Chicago, IL USA; 5 Faculté des Sciences, Université de Toliara, 601 Toliara, Madagascar; 6 Institut Pasteur (International Division), 25-28 Rue du Dr Roux, 75015 Paris, France; 7 Université de La Réunion, UMR Processus Infectieux en Milieu Insulaire Tropical (PIMIT), INSERM 1187, CNRS 9192, IRD 249, 97490 Sainte-Clotilde, La Réunion, France

**Keywords:** Bats, *Polychromophilus*, Eastern, MaxEnt, Madagascar

## Abstract

**Background:**

Numerous studies have been undertaken to advance knowledge of apicomplexan parasites infecting vertebrates, including humans. Of these parasites, the genus *Plasmodium* has been most extensively studied because of the socio-economic and public health impacts of malaria. In non-human vertebrates, studies on malaria or malaria-like parasite groups have been conducted but information is far from complete. In Madagascar, recent studies on bat blood parasites indicate that three chiropteran families (Miniopteridae, Rhinonycteridae, and Vespertilionidae) are infected by the genus *Polychromophilus* with pronounced host specificity: *Miniopterus* spp. (Miniopteridae) harbour *Polychromophilus melanipherus* and *Myotis goudoti* (Vespertilionidae) is infected by *Polychromophilus murinus*. However, most of the individuals analysed in previous studies were sampled on the western and central portions of the island. The aims of this study are (1) to add new information on bat blood parasites in eastern Madagascar, and (2) to highlight biotic and abiotic variables driving prevalence across the island.

**Methods:**

Fieldworks were undertaken from 2014 to 2016 in four sites in the eastern portion of Madagascar to capture bats and collect biological samples. Morphological and molecular techniques were used to identify the presence of haemosporidian parasites. Further, a MaxEnt modelling was undertaken using data from *Polychromophilus melanipherus* to identify variables influencing the presence of this parasite

**Results:**

In total, 222 individual bats belonging to 17 species and seven families were analysed. *Polychromophilus* infections were identified in two families: Miniopteridae and Vespertilionidae. Molecular data showed that *Polychromophilus* spp. parasitizing Malagasy bats form a monophyletic group composed of three distinct clades displaying marked host specificity. In addition to *P*. *melanipherus* and *P*. *murinus*, hosted by *Miniopterus* spp. and *Myotis goudoti*, respectively, a novel *Polychromophilus* lineage was identified from a single individual of *Scotophilus robustus*. Based on the present study and the literature, different biotic and abiotic factors are shown to influence *Polychromophilus* infection in bats, which are correlated based on MaxEnt modelling.

**Conclusions:**

The present study improves current knowledge on *Polychromophilus* blood parasites infecting Malagasy bats and confirms the existence of a novel *Polychromophilus* lineage in *Scotophilus* bats. Additional studies are needed to obtain additional material of this novel lineage to resolve its taxonomic relationship with known members of the genus. Further, the transmission mode of *Polychromophilus* in bats as well as its potential effect on bat populations should be investigated to complement the results provided by MaxEnt modelling and eventually provide a comprehensive picture of the biology of host-parasite interactions.

**Supplementary Information:**

The online version contains supplementary material available at 10.1186/s12936-021-03696-0.

## Background

In recent years, studies in different areas of the world have elucidated the biology, ecology, diversity, and evolutionary history of apicomplexan parasites infecting vertebrates [1–4]. Among these blood parasites, the genus *Plasmodium* has been the most intensively studied because of the millions of cases of malaria recorded per year, leading to more than 400,000 deaths annually in humans [5]. *Plasmodium* also infects different groups of vertebrates, such as reptiles, birds, and mammals, including bats [6–8]. The genus *Plasmodium* is now recognized to be polyphyletic and species diversification has led to the evolution of different malaria or malaria-related parasite lineages. For example, a recent study has highlighted that malarial parasites in general can be the subject of different host-switching events and shifts in life-history traits, which in turn insures their maintenance within hosts further facilitated by a simplification of their life cycle [8].

Based on previous observations, bats are also hosts of different malaria-related parasites, including nine genera of Haemoproteidae [8–15], but only four of these, including the genus *Polychromophilus*, are diagnosed based on molecular tools, which calls for an in-depth investigation of haemosporidian parasites, including their epidemiology, host range, distribution, transmission, and patterns of speciation.

With respect to the genus *Polychromophilus*, which is limited to bats, five species have been formerly identified using morphological characters (*Polychromophilus adami*, *Polychromophilus deanei*, *Polychromophilus corradetti*, *Polychromophilus melanipherus*, and *Polychromophilus murinus*) of which two have been molecularly characterized (*P*. *melanipherus* and *P*. *murinus*) infecting Miniopteridae [16, 17] and Vespertilionidae [17, 18], respectively. Further molecular research has revealed two additional taxa in Vespertilionidae hosts, classified as *Polychromophilus* species 1 in *Kerivoula hardwickii* from Cambodia [16] and *Polychromophilus* sp. 2 in *Pipistrellus grandidieri* (currently, *Pipistrellus* aff. *grandidieri* [19]) and *Laephotis capensis* from Guinea [10]. The remaining three species, *Polychromophilus adami*, *P*. *deanei*, and *P*. *corradetti* have only been diagnosed using morphological characters, and their taxonomic identity needs further molecular characterization.

On Madagascar, 46 species of bats are currently recognized with a level of endemism approaching 80 % (Goodman et al. pers. commun.). Bat species diversity in the eastern mesic portions of the island is lower than in the western dry zone [20, 21]. In terms of microorganisms hosted by Malagasy bats, different published studies have demonstrated the circulation of viruses, bacteria, and metazoan parasites in these nocturnal mammals [22–25]. Besides parasites of potential medical importance, bats from Madagascar also host malaria-related parasites, namely the genus *Polychromophilus* (Haemosporida: Plasmodiidae). Two species of *Polychromophilus* are currently documented in Malagasy bats: *P*. *melanipherus* infecting *Miniopterus* spp. and rarely *Paratriaenops furculus* (Rhinonycteridae) and *P. murinus* parasitizing *Myotis goudoti* (Vespertilionidae) [17, 26]. While 32 species of bats occurring in the western and the central portion of Madagascar were already screened for the presence of *Polychromophilus* [16, 17, 26], knowledge on *Polychromophilus* in bats occurring in the eastern area is not well-known and aspects on biotic and abiotic drivers of infection are far from complete. In the present study, *Polychromophilus* diversity and distribution in bats living in the oriental portion of the island was explored and the potential drivers of *Polychromophilus* infection across the island predicted based on new and previous records.

## Methods

### Study sites

**Fig. 1:**
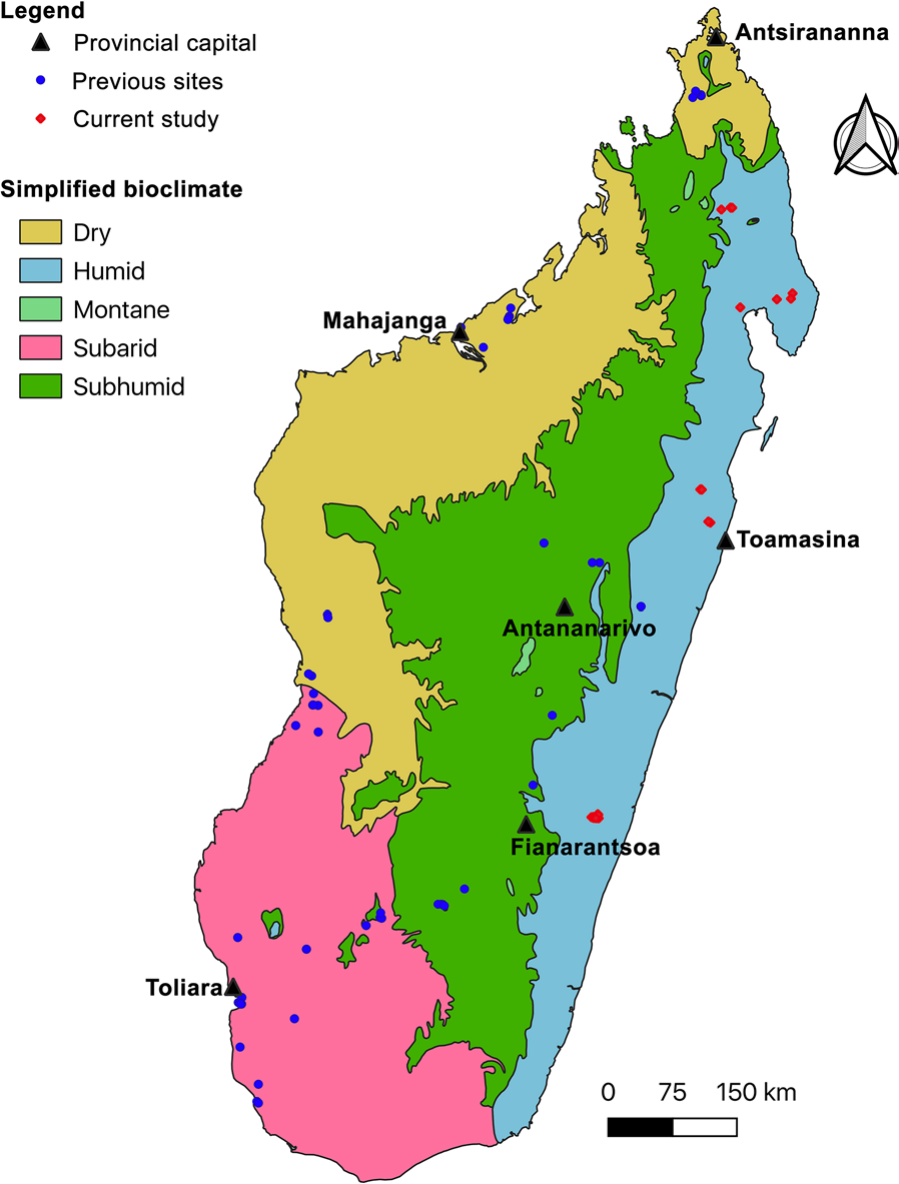
Localization of the different sites included in the current and previous studies

Fieldwork to sample bats was undertaken from 2014 to 2016 at four main sites in eastern Madagascar: Kianjavato Forest Station within the Paysage Harmonieux Protégé du Corridor Forestier Ambositra-Vondrozo and surrounding areas; Réserve Naturelle Intégrale de Betampona; Parc National de Masoala; and Parc National de Marojejy (Fig. 1, Additional file 1: Table S1). A range of habitat types were investigated, including day roosts in buildings, degraded areas associated with croplands, and lowland moist evergreen forests.

### Bat sampling

Bats were captured using mist nets and harp traps erected across flight pathways. Additionally, a hand net was used to capture bats inside day roost sites. Upon capture, each individual bat was placed separately in a clean cloth bag. Bat voucher specimens were morphologically identified and deposited at the Université d’Antananarivo, Mention Zoologie et Biodiversité Animale (formerly Département de Biologie Animale, UADBA), Antananarivo, and in the Field Museum of Natural History (FMNH), Chicago. The recent revision of Monadjem et al. [19] was followed for the taxonomy of certain Afro-Malagasy members of the family Vespertilionidae.

### Blood smear screening and prevalence of the infection

In the field, one thin blood smear per individual bat was prepared using a non-calibrated drop of blood. In the laboratory, blood smears were fixed with methanol, stained with Giemsa for 10 min at room temperature, rinsed with phosphate-buffered saline solution, and air-dried. Smears were subsequently examined using a Leica microscope (Leitz, Portugal) under 1000x magnification. For each blood smear, all monolayer fields were screened for the presence of haemosporidian parasites. The prevalence of the infection per species was obtained by dividing the number of infected individuals by the total number of individuals examined for each species [27, 28].

### DNA extraction, PCR amplification, and sequencing

In the field, blood spots from each individual bat were conserved on Whatman filter paper, air-dried, and stored at room temperature until DNA extraction. In the laboratory, two blood spots from microscopically positive samples were lysed in 0.5 % saponin solution during four hours at room temperature. Subsequently, they were washed with phosphate-buffered saline solution (PBS 1X). Total DNA extraction was performed using Instagene™ Matrix (BioRad, USA) following the manufacturer’s protocol. PCR amplification targeting the portion of mitochondrial Cytochrome *b* locus (Cyt *b*) of haemosporidian parasites was undertaken using a previously described nested PCR protocol [16, 18, 26] using Plas 1 and Plas 2 for the first PCR and Plas 3 and Plas 4 for the second PCR. PCR products were visualized in an electrophoresis gel and subsequently sent for Sanger sequencing to Genoscreen (Lille, France) using Plas 3 and Plas 4 primers.

### Phylogenetic analysis

Nucleotide sequences obtained from positive individual bats were manually edited using *Geneious* software [29]. Individual nucleotide sequences were subsequently aligned with other data on bat blood parasites downloaded from Genbank using MAFFT alignment implemented in *Geneious* software. Prior to determining the phylogenetic relationship of the parasites sequenced, the best substitution model was identified using jModelTest version 2.1.3 [30, 31] revealing GTR + I + G as the best substitution model. Subsequently, Bayesian inference consisting of two independent runs of four incremental Metropolis Coupled Markov Chain Monte Carlo (MC3) iterations starting from a random tree was conducted using MrBayes 3.1.2 [32]. This analysis consisted of two runs of 5,000,000 generations with trees and associated model parameters sampled every 500 generations. The first 25 % of the trees were discarded as a burn-in. New nucleotide sequences produced in the present study were deposited to Genbank under Accession numbers MW039207 to MW039233 (Additional file 2: Table S2).

### Statistical analysis and Maxent modelling of ***Polychromophilus***

A Generalized Linear Model (GLM) was constructed to investigate variation in *Polychromophilus* infection between *Miniopterus* spp. and sex classes using R version 3.0.0 [33]. Results of the GLM are expressed as adjusted odd-ratio with confidence interval at 95 %. The identification of variables controlling the distribution of *P. melanipherus* was conducted using the MaxEnt software version 3.3.3k [34]. Modelling was carried out using occurrence records obtained in the present study, as well as another recent study [26]. Environmental variables for each of the capture sites [34, 35] included the following nine bioclimatic variables: annual total evapotranspiration, maximum precipitation of the wettest month, minimum precipitation of the driest month, maximum temperature of the warmest month, minimum temperature of the coldest month, mean annual precipitation, mean annual temperature, numbers of months with a positive water balance, and annual water balance. These data were recovered at 30 arc seconds resolution for each site at [36]. Elevational data was downloaded from; Geology from the simplified geological map of Madagascar [37] and vegetation cover taken from Rakotondratsimba [38].

Model accuracy was assessed by splitting occurrence data into 70 % for training and 30 % for testing [34]. This approach was repeated five times. A set of 10,000 random points was used as background data to generate the Area under the Curve (AUC) [39, 40]. Models with an AUC above 0.8 were considered as informative [39, 41]. The output of MaxEnt modelling is presented in map form showing the probability of suitable and unsuitable area [34].

## Results

### Blood smear screening

In total, 222 bats belonging to 17 species were microscopically screened for the presence of haemosporidian parasites. Thirty-six individual bats representing six species from two families were found positive. Four of the six screened species of *Miniopterus* (Miniopteridae), as well as *Myotis goudoti* and *Scotophilus robustus* (Vespertilionidae), were positive for haemosporidian infection (Table 1). This is the first report of haemosporidian parasites in *S. robustus*, a species endemic to Madagascar. The prevalence of the infection ranged from 20 to 54.5 % in *Miniopterus* spp. (n = 88), 8 % in *Myotis goudoti* (n = 25), and 33 % in *Scotophilus robustus* (n = 3) (Table 1). Based on Generalized Linear Model analyses, the prevalence presented variation between species (p = 0.036) but not between sexes (p = 0.44). For the different positive species of *Miniopterus*, two, *Miniopterus gleni* (Adjusted Odd-Ratio: 2.17, CI 0.21–22.5) and *Miniopterus griveaudi* (Adjusted Odd-Ratio: 3.86, CI 0.35–43.05)—featured a high prevalence compared to the other species analysed.

### Phylogenetic analysis

**Fig. 2:**
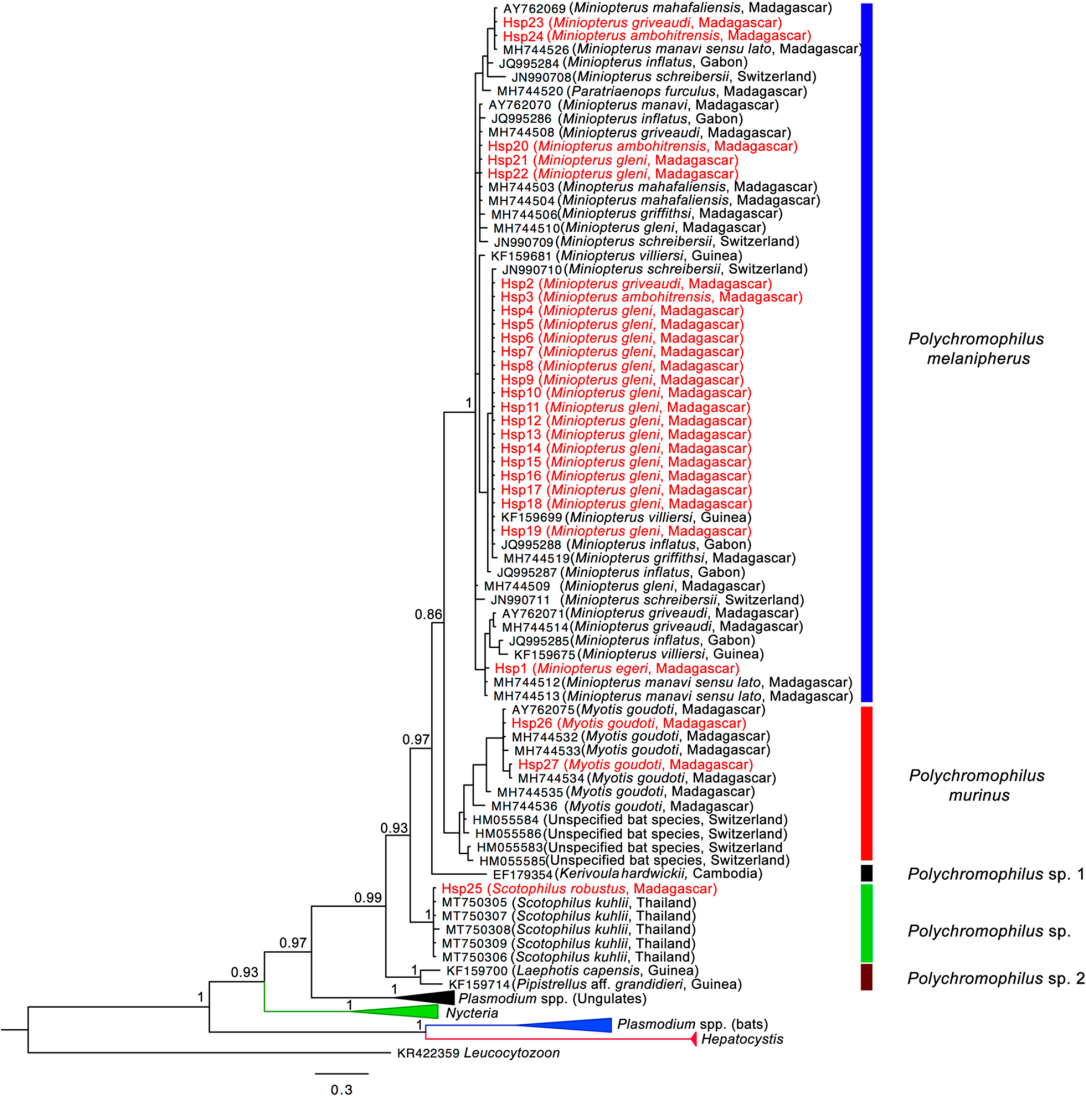
Phylogenetic relationship of haemosporidian parasites infecting bats based on Bayesian analysis. New sequences associated with this study are coloured in red. For the genus *Polychromophilus*, Isolate or Genbank accession number are followed by host species and locality

In total, 27 samples were amplified and sequenced. The Bayesian analysis based on 91 nucleotide sequences from Cyt *b* gene from this study, as well as those from different areas around the world, showed that the genus *Polychromophilus* forms a well-supported monophyletic clade (Fig. 2). Within this cluster, five branches are observed: *P*. *melanipherus* in members of the family Miniopteridae, *P*. *murinus* in Vespertilionidae, *Polychromophilus* sp. 1 in *Kerivoula hardwickii* from Cambodia, *Polychromophilus* sp. 2 in two species of Vespertilionidae (*Pipistrellus* aff. *grandidieri* and *Laephotis capensis*) from Guinea and a novel branch of *Polychromophilus*, annotated *Polychromophilus* sp. from *S. robustus* (Vespertilionidae) from Madagascar and *Scotophilus kuhlii* from Thailand. Nucleotide sequences of *Polychromophilus* from these two *Scotophilus* species present about 90 to 98 % of similarities (one nucleotide sequence from *Scotophilus* in Thailand (MT750308) was excluded in this comparison because of its reduced length). For Malagasy bats, nucleotide sequences obtained from those captured in the eastern portion of the island are composed of three different taxa: *P. melanipherus*, *P. murinus*, and the novel lineage of *Polychromophilus* (Fig. 2, Additional file 2: Table S2).

**Table 1:** *Polychromophilus* infection in bats from eastern Madagascar based on blood smear screening

Family	Species	N	Infected (in %)
Pteropodidae	*Rousettus madagascariensis*	25	0
Hipposideridae	*Macronycteris commersoni*	1	0
Emballonuridae	*Coleura kibomalandy*	9	0
	*Paremballonura atrata*	27	0
Myzopodidae	*Myzopoda aurita*	17	0
Molossidae	*Chaerephon atsinanana*	12	0
	*Mops leucostigma*	11	0
Vespertilionidae	*Myotis goudoti*	25	2 (8)
	*Laephotis matroka*	2	0
	*Pipistrellus raceyi*	2	0
	*Scotophilus robustus*	3	1 (33)
Miniopteridae	*Miniopterus ambohitrensis*	9	3 (33)
	*Miniopterus brachytragos*	6	0
	*Miniopterus* cf. *aelleni*	3	0
	*Miniopterus egeri*	5	1 (20)
	*Miniopterus gleni*	43	17 (39.5)
	*Miniopterus griveaudi*	22	12 (54.5)
	Total	222	36 (16.2)

*N* number of individuals analyzed

### Distribution of *Polychromophilus*spp. on Madagascar

**Fig. 3:**
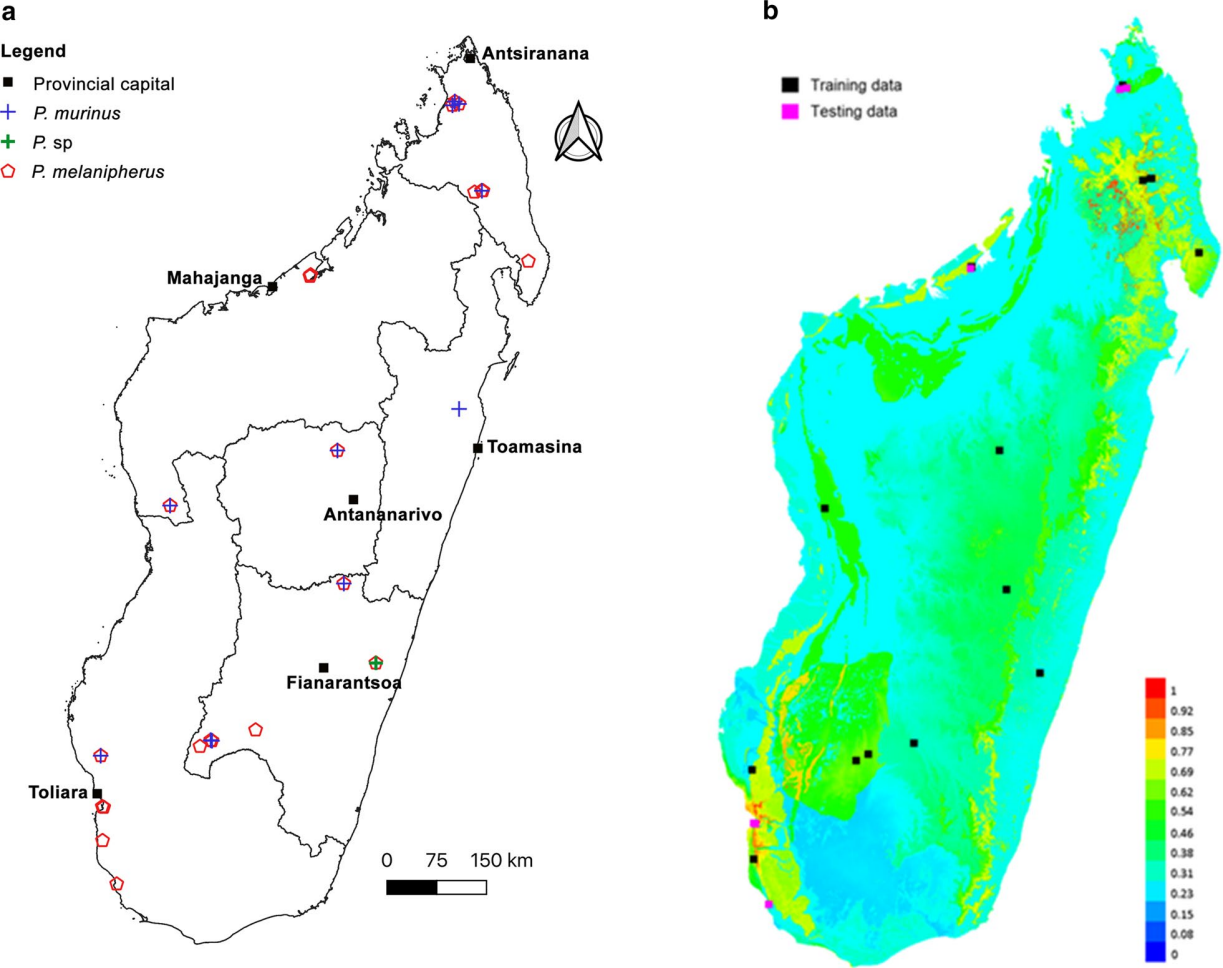
**a** Distribution of *Polychromophilus* spp. on Madagascar. The different delineated areas represent provincial boundaries.** b** Predicted occurrence of *Polychromophilus melanipherus* on Madagascar based on MaxEnt analysis

Of the seven bat families investigated from Malagasy samples in the current and previous studies, *Polychromophilus* spp. infect mainly two bat families, Miniopteridae and Vespertilionidae, with *P. melanipherus* found in members of the family Miniopteridae (10 species infected), *P. murinus* parasitizing a member of the family Vespertilionidae (*Myotis goudoti*), and *Polychromophilus* sp. found in another vespertilionid (*Scotophilus robustus*) sampled at Kianjavato (Fig. 3 a, b).

### *Polychromophilus* sp.

*Polychromophilus* sp., the novel lineage, was identified in *Scotophilus robustus* based on morphological and molecular screening, but little can be said about its distributional range in Madagascar due to the single known occurrence.

### Polychromophilus murinus

*Polychromophilus murinus* was restricted to *Myotis goudoti* sampled at different sites across the island. *Polychromophilus murinus* was present in 11 out of the 17 sampled sites and based on current data is specific to *Myotis goudoti* (Additional file 3: Table S3)

### Polychromophilus melanipherus

*Polychromophilus melanipherus*, associated with the genus *Miniopterus*, is a widespread parasite across the Old World distribution of the genus. Based on previous studies and data presented herein, *P. melanipherus* was identified from 23 out of the 28 sites sampled in Madagascar, including a range of different habitat types (Additional file 3: Table S3), this information was used in a MaxEnt analysis to model the parasites’ presence on the island. The model had an AUC of 0.855 and the main four variables predicting suitable habitat on the presence of *Polychromophilus* were vegetation (61.1 %), geology (29.4 %), annual water balance (4.9 %), and annual total evapotranspiration (4.6 %). The remaining eight variables were less explicative (percent contribution between 0 and 1.3 %) and were omitted from the final model.

MaxEnt prediction indicated an area of highest environmental suitability in the northern and southwestern areas of Madagascar, corresponding to orange to red colours (Fig. 3b), with a probability of infection ranging from 0.7 to 1. Further, *P. melanipherus* may occur in diverse areas with different environmental conditions, corresponding to the green colour and a probability of infection ranging from 0.54 to 0.69 (Fig. 3b). Based on recent vegetation classification of Madagascar, regions of highest suitability for *Polychromophilus* included northern moist evergreen forest, southwestern dry spiny thicket, and western dry deciduous forest. It appears that in Madagascar *P. melanipherus* is tightly associated with the presence of *Miniopterus* spp.

## Discussion

### ***Polychromophilus*** infection in Malagasy bats and phylogenetic analyses

On the basis of the results presented herein including the analysis of seven different families of bats, *Polychromophilus* infection in Malagasy bats from the eastern portion of the island appears to be limited to two families (Miniopteridae and Vespertilionidae), while the other five tested families (Pteropodidae, Hipposideridae, Emballonuridae, Myzopodidae, and Molossidae) were negative. It is also known from Malagasy members of the family Rhinonycteridae (see below). Such host-parasite associations are congruent with previous investigations carried out in different portions of the world [10, 17, 18, 26, 42]. To date, 10 out of the 12 species of *Miniopterus* tested and occurring on Madagascar are infected by *P*. *melanipherus* based on morphological or molecular screenings. *Miniopterus brachytragos* was represented by six individuals in the current study, which were all negative. Hence, the absence of *P. melanipherus* infection in *M*. *brachytragos* may be associated with the limited sample size, as this species was previously found infected by Haemosporidae [43]. *Miniopterus petersoni*, with a limited geographical distribution [44], has not yet been analysed to date due to the absence of blood samples. Based on GLM analysis, *M. gleni* and *M. griveaudi* present higher risk of infection than the other Malagasy species within this genus. This may be due to their roosting behaviour, as these two species occur in large day roosting colonies within caves and both species have broad geographical distributions and little genetic variability, which can be related to their high dispersal capacity [44–46].

Phylogenetic analysis based on a portion of Cyt *b* gene of *Polychromophilus* spp. in bats from different localities showed that they form a monophyletic group composed of five distinct clades, of which three are documented on Madagascar. *Polychromophilus melanipherus* was identified in *Miniopterus* spp., providing further support that this species of blood parasite being specific to the Miniopteridae, which has a broad Old World distribution [8, 10, 47]. This parasite infects different species of *Miniopterus* regardless of presumed geographic or host species barriers [10, 17, 42, 48]. Apart from *Miniopterus* spp., *Paratriaenops furculus* (Rhinonycteridae) has already been reported to be infected with *Polychromophilus melanipherus* [26], whereas a closely related member of this family, *Triaenops menamena*, which occurs in the same day roost sites as *Paratriaenops furculus* [44, 49] tested negative (n = 42) for this parasite [26].

The second clade represented by *Polychromophilus murinus*, occurred in different genera and species of the family Vespertilionidae [26, 50, 51]. Compared to infection of *P*. *murinus* in European bats, based on current data from Malagasy Vespertilionidae, only *Myotis goudoti* is infected by *P*. *murinus*. A third lineage of *Polychromophilus*, identified herein as *Polychromophilus* sp. based on molecular data, was found in a single specimen of *Scotophilus robustus* (Vespertilionidae). The phylogenetic tree shows that this last lineage falls within the same cluster as sequences from *S. kuhlii* from Thailand [52] representing an undescribed *Polychromophilus* taxon. With one positive specimen among three captured individuals of *S. robustus* and five sequences from *Scotophilus* from Thailand, it would appear that this parasite may be strictly associated with the genus *Scotophilus* spp.; further samples and analyses are needed to resolve the taxonomy of *Polychromophilus* sp. based on morphological and molecular diagnosis.

The other two *Polychromophilus* clusters identified in Fig. 2 as *Polychromophilus* sp. 2 in *Laephotis capensis* and *Pipistrellus* aff. *grandidieri* from West Africa, and *Polychromophilus* sp. 1 in *Kerivoula hardwickii* from southeast Asia [10, 16] also need further investigation to have additional information on their taxonomy. To date, 38 out of the 46 species of bats occurring on Madagascar have been screened for the presence of apicomplexan parasites. The balance of eight species needs to be examined as material becomes available. Interestingly, *Plasmodium* from ungulates (as exemplified by *P*. *bubalis* and *P. odocoilei*) are closely related to *Polychromophilus* from bats.

### Distribution of* Polychromophilus* spp. in Madagascar

Data on *P. melanipherus* was used to model the distribution of this bat blood parasite in members of the genus *Miniopterus*, as these bats are widespread on Madagascar from sea level to up to 1800 m [44]. Data from Raharimanga et al. [43] was not included in the modelling analysis presented herein as no molecular haematoparasite data was available for species determination. In total, 10 out of the 11 *Miniopterus* spp. screened for the presence of *P. melanipherus* were positive. Members of the family Miniopteridae use a range of roost sites such as caves, crevices or rock overhangs [44, 49] and generally live in mixed colonies with other species, often *Myotis goudoti* [44]. The dispersal capacity for certain members of the genus on Madagascar with broad geographic distributions is notably high, as exemplified by *Miniopterus gleni* and *M*. *griveaudi* [44, 49]. *Polychromophilus melanipherus* appears to be a cosmopolitan blood parasite of *Miniopterus* spp. across the Old World.

Based on the Maxent analysis, vegetation and geology are the two most significant parameters and contributed to more than 90 % of the variables explaining the occurrence of *P. melanipherus* on Madagascar. The vegetation variable showed a notable difference between the western and eastern portions of the island. In the west, three main vegetation types occur, including dry forest in the north, dry deciduous forest in the central area, and dry spiny thicket in the southwest. These vegetational gradients are important in the ecology of *Miniopterus* spp. associated with their feeding behaviour at a local scale and their dispersal at a broader geographical scale. In addition, throughout much of the west exposed limestone formations occur and often with extensive cave systems where *Miniopterus* spp. roost in sympatry or in syntopy within the cave. At such day roost sites, *Miniopterus*, as well as other bat species, most notably *Myotis goudoti*, occur in large to moderately large mono- or multi-specific groups, which may favour parasite exchange.

The analysis indicates that the eastern and central portions of Madagascar, characterized by natural moist evergreen forests and mesic climatic conditions, are less favourable for *Miniopterus*. Also, the local geology of this area lacks exposed sedimentary rock and deep cave formations; this results in bat day roost sites being limited to crevices or tree holes and smaller roosting groups. While *P. melanipherus* is associated with the genus *Miniopterus*, several abiotic variables help explain the lower prevalence of this parasite in the eastern portion of the island. It is important to mention that *P. melanipherus* and *P. murinus* generally co-occur within the same locality as their respective hosts sometimes live in sympatry or in syntopy within their day roost sites but no case of co-infection in a single individual is known.

### Drivers of ***Polychromophilus ***infection in bats

Dick and Dittmar [53] hypothesized that the type of day roost site, colony size, and bioclimatic aspects can affect the exposure of bats to insect vectors, and, hence, parasite transmission. Based on the results obtained herein and data from the literature, on Madagascar *Polychromophilus* infection is limited to several species from three different families of bats, and some inferences based on the analyses presented herein can be presented about potential drivers of *Polychromophilus* infection. To this end, it is possible that *Polychromophilus* infection in bats is driven by two main factors: the ecological niche they occupy and the behavioural aspects of bat hosts and the presence of competent vectors.

### Ecological niche and bat behaviour

The three *Polychromophilus* taxa identified in Malagasy bats show a certain level of host specificity that may be related to the ecological niche and behaviour of their bat hosts. As *P. melanipherus* infects almost all *Miniopterus* spp. on Madagascar, hypotheses regarding parasites maintenance and transmission can be suggested. The genus *Miniopterus* occurs in a wide range of habitats on the island with some of the species forming large monospecific or multispecific colonies. *Miniopterus gleni* and *M. griveaudi* often live in caves and rock shelters located near freshwater streams or in areas protected from solar radiation and with local humid conditions. Further, these two bat species often share or occur in relatively close proximity to other cave roosting bat species. *Miniopterus gleni* is the only species with a large distribution across much of the island, from sea level to 1200 m, and can also co-occur with other members of the genus [49]. Further, phylogeographic studies show little genetic variation across the different localities it is known from and it can be presumed to disperse widely [45]. The role of this species as a bridge for *Polychromophilus* transmission is therefore probable. Nonetheless, this supposition needs further consideration with regards to the ecology, ectoparasites, and behaviour of this bat species.

*Polychromophilus murinus* on Madagascar is only known from *Myotis goudoti*. This endemic bat species shows little phylogeographical structure, best explained by broad dispersal [54]. *Myotis goudoti* occurs in various habitat types on the island and small to large day roosts are known from caves, crevices, and tree holes [44]. Although this species often occurs in syntopy in the same day roost site with small or middle-sized *Miniopterus*, such as *M. griveaudi*, *M. ambohitrensis*, or *M*. *majori*, no co-infection of *P. murinus* has been reported and best interpreted as the occurrence of distinct transmission filters between genera/families of bats.

### Presence of competent vectors and fly/bat specificity

*Polychromophilus* has been proposed to be transmitted by bat flies (Diptera: Hippoboscoidea: Nycteribiidae) [12], which are highly specialized ectoparasites [55–58]. Nycteribiid flies and their associated bat hosts show a strong association providing the means for parasite transmission through their respective life cycles. As a vector-borne infection, the transmission of *Polychromophilus* is presumably insured by the presence of competent vectors that are tightly associated with bat hosts. The molecular screening of 38 individual nycteribiids belonging to three species (*Penicillidia leptothrinax*, *Penicillidia* sp. cf. *fulvida*, and *Nycteribia stylidiopsis*) revealed the presence of *Polychromophilus* spp. [26]. While no detailed work on the role of Nycteribiidae as vector of *Polychromophilus* has been conducted on Madagascar, three species of Nycteribiidae, namely *Penicillidia leptothrinax*, *Nycteribia stylidiopsis* and *Penicillidia* sp. are local candidate vectors of *Polychromophilus* [26]. *Penicillidia leptothrinax* and *N. stylidiopsis* have been previously reported positive for *Polychromophilus melanipherus.* These positive bat flies included four *Penicillidia leptothrinax* specimens (two sampled on *Miniopterus aelleni* and two on *M. manavi sensu lato*), in addition to a single *N. stylidiopsis* obtained from *M. gleni*. These two bat fly species are common on bats of the genus *Miniopterus* and can be putatively considered as vectors of *Polychromophilus melanipherus* [26, 57]. As far as *P. murinus* is concerned, one specimen of *Penicillidia* sp. was found positive and collected on a *Miniopterus* individual that was negative to *Polychromophilus melanipherus*, and this result probably indicates considerable ectoparasite exchange between members of the genera *Miniopterus* and *Myotis*, which is supported by three shared species of nycteribiids (*N. stylidiopsis*, *Penicillidia decipiens*, and *P. leptothrinax*) (Dick, pers. commun.). Based on available information, it can be assumed that these three species of Nycteribiidae are candidate vectors of *Polychromophilus* spp. in Malagasy bats. Given the lack of host specificity in nycteribiid flies between Malagasy bats of the genera *Miniopterus* and *Myotis* and the proposed role of these ectoparasites in *Polychromophilus* infection, it might be assumed that there would be less host specificity in *P. murinus* and *P. melanipherus*. However, this is not the case and some other form of filter seems to be in place to reduce cross-genera infection. In contrast, *Polychromophilus* sp. is only known to date from *Scotophilus robustus*, which is the host for a different genus of nycteribid (*Basilia*) never collected on Malagasy *Miniopterus* or *Myotis*.

## Conclusions

Across the Old World, *Polychromophilus melanipherus* infection is widespread in *Miniopterus* spp. (family Miniopteridae). Malagasy members of the family Vespertilionidae are infected by two different species of *Polychromophilus*. Based on current findings, three taxa of *Polychromophilus* are currently known in bat species from the island. While *P. melanipherus* and *P. murinus* appeared to be broadly distributed within their identified hosts namely *Miniopterus* spp. and *Myotis goudoti*, respectively, the third identified *Polychromophilus* taxon represents a new lineage. According to the data presented herein, *Polychromophilus* is closely related with specific hosts (species or families) and their occurrence is linked with the abiotic factors as exemplified by the MaxEnt analysis of *P. melanipherus*, as well as the presence of nycteribiid vectors favouring parasite transmission. Although the majority of Malagasy bat species have now been examined for the presence of *Polychromophilus* spp. using morphological and/or molecular screening, additional work is needed on the few non-analysed species and to increase sample sizes for some others that have not tested positive. Future studies should focus on the life cycle of *Polychromophilus* spp., and their evolutionary history and potential adverse effect on the bat hosts. Further, the importance of nycteribiid flies in the transmission of the different species of *Polychromophilus* in bats from Madagascar should be investigated.

## Supplementary Information

**Additional file 1: Table S1.** Localities sampled in the previous and present study.

**Additional file 2: Table S2.** Genbank accession numbers of blood parasites included in the present study. Isolates and their Genbank accession number produced in the frame of the present work are highlighted in bold.* FMNH* Field Museum of Natural History,* UADBA* Université d’Antananarivo, Département de Biologie Animale.

**Additional file 3: Table S3.** Localities with positive occurrence of Polychromophilus in Madagascar including the origin of the sample (cave or trapping) and their location (latitude, longitude).

## Data Availability

All data generated or analysed during this study are included in this article.
