# Supplementary material for: Diversity, distribution, and drivers of polychromophilus infection in Malagasy bats
Source: Malar J. 2021 Mar 20;20:157. doi: 10.1186/s12936-021-03696-0 (PMC7980569; doi:10.1186/s12936-021-03696-0)
Supplement: Supplementary file 1 — Additional file 1: Table S1. Localities sampled in the previous and present study. [file 12936_2021_3696_MOESM1_ESM.docx]

Table S1. Localities sampled in the previous and present study.

| **Site n#** | **Source** | **Locality** | Latitude | Longitude |
| --- | --- | --- | --- | --- |
| 1 | [27] | Réserve Spéciale d’Ambohitantely, Grotte des Chauves-souris | 18.181167°S | 47.2893°E |
| 2 | [27] | Réserve Spéciale d’Ambohitantely, début sentier touristique | 18.181167°S | 47.2893°E |
| 3 | [27] | Sous-préfecture d’Anjozorobe, Forêt d’Antsahabe, Andohasahabe | 18.410033°S | 47.93678°E |
| 4 | [27] | Ambohibeloma, 3.2 km W d’Anjozorobe | 18.40882°S | 47.85168°E |
| 5 | [27] | Parc National d’Ankarana, Ambahibe Cave, 2 km W Mahamasina | 12.967667°S | 49.12052°E |
| 6 | [27] | Parc National d’Ankarana, Grotte des Chauves-souris, 3 km NW Mahamasina | 12.956317°S | 49.11808°E |
| 7 | [27] | Parc National d’Ankarana, Grotte d’Andrafiabe, 3.3 ESE d’Andrafiabe | 12.931667°S | 49.06°E |
| 8 | [27] | Parc National d’Ankarana, 2.6 km E Andrafiabe, in forest near Andrafiabe Cave | 12.931667°S | 49.05667°E |
| 9 | [27] | Parc National d’Ankarana, Grotte du troisième Canyon, along Andokotokana River | 12.91417°S | 49.055°E |
| 10 | [27] | Parc National d’Ankarana, 2.2 km ESE Amboandriky, Grotte d’Ambatoharanana | 12.9883°S | 49.0217°E |
| 11 | This study | Parc National Masoala, Sarahandrano | 15.27106°S | 50.18392°E |
| 12 | This study | Parc National Masoala, Sahabe | 15.33373°S | 50.16545°E |
| 13 | This study | Parc National Marojejy, Antsahabe | 14.27393°S | 49.4657°E |
| 14 | This study | Parc National Marojejy, Beamalona | 14.27086°S | 49.46233°E |
| 15 | This study | Parc National Marojejy, Mandena | 14.27344°S | 49.48093°E |
| 16 | This study | Parc National Marojejy, Antanimbaribe | 14.29427°S | 49.3545°E |
| 17 | [27] | Commune rurale d’Ankily, west of Ihosy off RN 7 | 22.385°S | 46.09556°E |
| 18 | [27] | Grotte d’Andranomilitra, west of Ihosy off RN 7 | 22.38524°S | 46.05601°E |
| 19 | [27] | Grotte de Fandanana, 4.1 km NE de Fandriana | 20.182983°S | 47.38552°E |
| 20 | [27] | Parc National d’Isalo, 3.8 km NW de Ranohira, along Namaza River | 22.54°S | 45.38°E |
| 21 | [27] | Parc National d’Isalo, Grotte de Bekapity | 22.633336°S | 45.21808°E |
| 22 | [27] | Edge of Parc National of Isalo, 7.8 km N Ranohira, along Menamaty River | 22.548°S | 45.399°E |
| 23 | [27] | Parc National de l’Isalo, Ambinanindranohira-bas, Andranomboalavo | 22.4858°S | 45.3868°E |
| 24 | [27] | Zazafotsy | 22.20708°S | 46.36339°E |
| 25 | [27] | Ihosy, Bureau du chef de la Région, | 22.40336°S | 46.12887°E |
| 26 | [27] | Vohiposa, CSB II | 20.99598°S | 47.16156°E |
| 27 | This study | Kianjavato Forest Station, Grotte Andoharano | 21.37341°S | 47.86042°E |
| 28 | This study | Kianjavato Forest Station, Station FOFIFA | 21.37904°S | 47.86769°E |
| 29 | This study | Kianjavato Forest Station, Site 2 | 21.37911°S | 47.86901°E |
| 30 | This study | Kianjavato Forest Station, Grotte Ampatsakana | 21.37724°S | 47.87723°E |
| 31 | This study | Kianjavato Forest Station, Grotte Sangasanga | 21.37512°S | 47.85926°E |
| 32 | This study | Kianjavato Forest Station, Site 1 | 21.37904°S | 47.86769°E |
| 33 | This study | Kianjavato Forest Station, Site 3 | 21.38061°S | 47.90532°E |
| 34 | This study | Kianjavato Forest Station, Grotte Ankazotokana | 21.37065°S | 47.8416°E |
| 35 | This study | Kianjavato Forest Station, Site 5 | 21.38243°S | 47.89855°E |
| 36 | This study | Kianjavato Forest Station, Site 2 | 21.37911°S | 47.86907°E |
| 37 | This study | Kianjavato Forest Station, Station FOFIFA | 21.37854°S | 47.86769°E |
| 38 | This study | Kianjavato Forest Station, Station FOFIFA | 21.37854°S | 47.86749°E |
| 39 | This study | Kianjavato Forest Station, Grotte near Sangasanga | 21.36787°S | 47.86319°E |
| 40 | This study | Kianjavato Forest Station, Proximity of site 3 | 21.38061°S | 47.90532°E |
| 41 | This study | Kianjavato Forest Station, Grotte mavogisy | 21.3782°S | 47.90153°E |
| 42 | This study | Kianjavato Forest Station, Grotte Seranantsara | 21.36421°S | 47.88977°E |
| 43 | This study | Kianjavato Forest Station, Ambolotara | 21.38316°S | 47.92598°E |
| 44 | This study | Kianjavato Forest Station, Morarano | 21.33549°S | 47.91588°E |
| 45 | This study | Kianjavato Forest Station, Ambinany Lalangy | 21.34209°S | 47.92178°E |
| 46 | This study | Kianjavato Forest Station, Ambinany Lalangy | 21.34252°S | 47.92141°E |
| 47 | This study | Kianjavato Forest Station, Ambalahosy | 21.38177°S | 47.89281°E |
| 48 | [27] | Ambovondramanesy village near Berivotra, along RN 4 | 15.9°S | 46.5833°E |
| 49 | [27] | Mahajanga, Petite Plage | 15.66797°S | 46.32205°E |
| 50 | [27] | Grotte d’Anjohikely (south entrance), 1.5 km NE d’Antanamarina | 15.560883°S | 46.8742°E |
| 51 | [27] | Grotte d’Anjohibe, 3.7 km NE d’Antanamarina | 15.53815°S | 46.88598°E |
| 52 | [27] | Grotte d’Anjohikely 2, 1.6 km NE d’Antanamarina | 15.5589°S | 46.8775°E |
| 53 | [27] | Cascade d’Antanamarina | 15.576183°S | 46.86928°E |
| 54 | [27] | Grotte de Beenta, 2 km W de Mitsinjo | 15.44251°S | 46.90207°E |
| 55 | [27] | Parc National de Bemaraha, Anjohikinakina 15.5 km N de Bekopaka | 19.0099°S | 44.7677°E |
| 56 | [27] | Limit of Parc National de Bemaraha, Ankapoka | 19.0449°S | 44.7735°E |
| 57 | [27] | Andasibe, CEG | 18.92016°S | 48.41768°E |
| 58 | [27] | Outskirts of Andasibe, Mangarivotra, Ambany Atsinanana | 18.91984 | 48.42044°E |
| 59 | This study | Réserve Naturelle Intégrale de Betampona, Rendrirendry Betampona | 17.9311°S | 49.2034°E |
| 60 | This study | Réserve Naturelle Intégrale de Betampona, Fotsimavo | 17.9433°S | 49.227°E |
| 61 | This study | Réserve Naturelle Intégrale de Betampona, Ambodirafia | 17.55677°S | 49.10896°E |
| 62 | This study | Réserve Naturelle Intégrale de Betampona, RNI Betampona | 17.55828°S | 49.12198°E |
| 63 | This study | Réserve Naturelle Intégrale de Betampona, RNI Betampona | 17.5567°S | 49.12156°E |
| 64 | This study | Réserve Naturelle Intégrale de Betampona, RNI Betampona | 17.55417°S | 49.11971°E |
| 65 | This study | Réserve Naturelle Intégrale de Betampona, Rendrirendry Betampona | 17.931°S | 49.203°E |
| 66 | This study | Réserve Naturelle Intégrale de Betampona, Fotsimavo | 17.55916°S | 49.12732°E |
| 67 | This study | Parc National Masoala, Ambohitsitondroina | 15.34097°S | 50.00123°E |
| 68 | This study | Parc National Masoala, Tampolo | 15.43456°S | 49.57494°E |
| 69 | [27] | Grotte de Sarodrano (sea cave) | 23.53°S | 43.73°E |
| 70 | [27] | Grotte de Makis (Mikea), near Hotel la Mangrove on Toliara-St. Augustin Road | 23.47211°S | 43.77069°E |
| 71 | [27] | Grotte de Bekoaky, 9.4 km SSE d’Ankililaoka | 22.773°S | 43.72267°E |
| 72 | [27] | Grotte de Tanambao (Bishiko), 0.75 km E de St. Augustin | 23.54888°S | 43.7674°E |
| 73 | [27] | Grotte d'Ambanilia, 3.7 km SSE de Sarodrano | 23.53995°S | 43.74605°E |
| 74 | [27] | St. Augustin, in lycée building | 23.5493°S | 43.7572°E |
| 75 | [27] | Parc National de Tsimanampetsotsa, Grotte d’Andranoilovy | 24.05°S | 43.75°E |
| 76 | [27] | Grotte d’Androimpano, 4.2 km NE d’Itampolo (village), on old road to Ejeda | 24.48353°S | 43.96328°E |
| 77 | [27] | Itampolo (village) | 24.68431°S | 43.94583°E |
| 78 | [27] | Grotte de Vintane (Vintany), 4.1 km SE d’Itampolo | 24.70236°S | 43.96372°E |
| 79 | [27] | Betioky Sud, New Lutheran Church | 23.71986°S | 44.3835°E |
| 80 | [27] | Sakaraha, Direction des Eaux et forêts, Bureau chef de cantonnement | 22.90946°S | 44.52279°E |
| 81 | [27] | Sakaraha, near Direction des Eaux et forêts complex at edge of town | 22.909°S | 44.523°E |
| 82 | [27] | Marofandilia (village), Ecole primaire | 20.06743°S | 44.658°E |
| 83 | [27] | Kirindy (village) | 20.06332°S | 44.59679°E |
| 84 | [27] | Antanandava, Eglise FLM, 5.8 km NE de Beroboka Sud | 19.92973°S | 44.60594°E |
| 85 | [27] | Tsimafana, CEG de Tsimafana | 19.7235°S | 44.58432°E |
| 86 | [27] | 0.8 km N de Kirindy (village) | 20.06222°S | 44.60126°E |
| 87 | [27] | Marofototra, Tsarafototra FLM | 20.30244°S | 44.39807°E |
| 88 | [27] | Mahabo, EPP de Mahabo | 20.3777°S | 44.66072°E |
| 89 | [27] | Belo Tsiribihina, Central Hospital | 19.7004°S | 44.5475°E |
