# Supplementary material for: Diversity, distribution, and drivers of polychromophilus infection in Malagasy bats
Source: Malar J. 2021 Mar 20;20:157. doi: 10.1186/s12936-021-03696-0 (PMC7980569; doi:10.1186/s12936-021-03696-0)
Supplement: Supplementary file 2 — Additional file 2: Table S2. Genbank accession numbers of blood parasites included in the present study. Isolates and their Genbank accession number produced in the frame of the present work are highlighted in bold. FMNH Field Museum of Natural History, UADBA Université d’Antananarivo, Département de Biologie Animale. [file 12936_2021_3696_MOESM2_ESM.docx]

Table S2. Genbank accession numbers of blood parasites included in the present study. Isolates and their Genbank accession number produced in the frame of the present work are highlighted in bold. FMNH = Field Museum of Natural History, UADBA = Université d’Antananarivo, Département de Biologie Animale,

| **Isolate** | **Genbank number** | **Host species** | **Museum vouvher** | **Origin** |
| --- | --- | --- | --- | --- |
|  | AY762069 | *Miniopterus mahafaliensis* | FMNH 172924 | Madagascar |
|  | KF159695 | *Epomophorus gambianus* |  | Guinea |
|  | KF159701 | *Epomops buettikoferi* |  | Guinea |
|  | KR422359 | *Falco sparverius* |  | United states |
|  | EF179355 | *Megaderma spasma* |  | Cambodia |
|  | KF159720 | *Rhinolophus alcyone* |  | Côte d'Ivoire |
|  | KF159690 | *Rhinolophus landeri* |  | Guinea |
|  | KF159710 | *Hipposideros cyclops* |  | Liberia |
|  | KF159716 | *Hipposideros cyclops* |  | Liberia |
|  | KF159674 | *Hipposideros cyclops* |  | Liberia |
|  | KF159671 | *Myonycteris angolensis* |  | Guinea |
|  | KF159692 | *Myonycteris angolensis* |  | Guinea |
|  | MG709243 | *Odocoileus virginianus* |  | USA |
|  | MG709246 | *Odocoileus virginianus* |  | USA |
|  | MH177860 | *Odocoileus virginianus* |  | USA |
|  | MN688306 | *Buffalo bubalis* (water buffalo) |  | Thailand |
|  | MN688307 | *Buffalo bubalis* (water buffalo) |  | Thailand |
|  | MN688308 | *Buffalo bubalis* (water buffalo) |  | Thailand |
|  | MN688309 | *Buffalo bubalis* (water buffalo) |  | Thailand |
|  | MN688310 | *Buffalo bubalis* (water buffalo) |  | Thailand |
| 36MG | MH744503 | *Miniopterus mahafaliensis* | FMNH 217957 | Madagascar |
| 744MG | MH744504 | *Miniopterus mahafaliensis* | FMNH 218041 | Madagascar |
| 169MG | MH744506 | *Miniopterus griffithsi* | UADBA SMG 17447 | Madagascar |
| 545MG | MH744508 | *Miniopterus griveaudi* | FMNH 221345 | Madagascar |
| 775MG | MH744509 | *Miniopterus gleni* | UADBA 50170 | Madagascar |
| 781MG | MH744510 | *Miniopterus gleni* | FMNH 218032 | Madagascar |
| 51MG | MH744512 | *Miniopterus manavi sensu lato* | FMNH 221423 | Madagascar |
| 53MG | MH744513 | *Miniopterus manavi sensu lato* | FMNH 221429 | Madagascar |
| 437MG | MH744514 | *Miniopterus griveaudi* | UADBA 33962 | Madagascar |
| 183MG | MH744519 | *Miniopterus griffithsi* | UADBA SMG 17464 | Madagascar |
| 57MG | MH744520 | *Paratriaenops furculus* | UADBA SMG 17186 | Madagascar |
| 52MG | MH744526 | *Miniopterus manavi sensu lato* | FMNH 221425 | Madagascar |
|  | AY762071 | *Miniopterus griveaudi* | FMNH 172862 | Madagascar |
| **Hsp1** | **MW039207** | *Miniopterus egeri* | MR15 | Madagascar |
| **Hsp2** | **MW039208** | *Miniopterus griveaudi* | MR85 | Madagascar |
| **Hsp3** | **MW039209** | *Miniopterus ambohitrensis* | MR163 | Madagascar |
| **Hsp4** | **MW039210** | *Miniopterus gleni* | MR164 | Madagascar |
| **Hsp5** | **MW039211** | *Miniopterus gleni* | MR179 | Madagascar |
| **Hsp6** | **MW039212** | *Miniopterus gleni* | MR180 | Madagascar |
| **Hsp7** | **MW039213** | *Miniopterus gleni* | MR184 | Madagascar |
| **Hsp8** | **MW039214** | *Miniopterus gleni* | MR212 | Madagascar |
| **Hsp9** | **MW039215** | *Miniopterus gleni* | MR216 | Madagascar |
| **Hsp10** | **MW039216** | *Miniopterus gleni* | MR217 | Madagascar |
| **Hsp11** | **MW039217** | *Miniopterus gleni* | MR219 | Madagascar |
| **Hsp12** | **MW039218** | *Miniopterus gleni* | MR222 | Madagascar |
| **Hsp13** | **MW039219** | *Miniopterus gleni* | MR223 | Madagascar |
| **Hsp14** | **MW039220** | *Miniopterus gleni* | MR226 | Madagascar |
| **Hsp15** | **MW039221** | *Miniopterus gleni* | MR229 | Madagascar |
| **Hsp16** | **MW039222** | *Miniopterus gleni* | MR230 | Madagascar |
| **Hsp17** | **MW039223** | *Miniopterus gleni* | MR233 | Madagascar |
| **Hsp18** | **MW039224** | *Miniopterus gleni* | MR234 | Madagascar |
| **Hsp19** | **MW039225** | *Miniopterus gleni* | MR221 | Madagascar |
| **Hsp20** | **MW039226** | *Miniopterus ambohitrensis* | MR173 | Madagascar |
| **Hsp21** | **MW039227** | *Miniopterus gleni* | MR231 | Madagascar |
| **Hsp22** | **MW039228** | *Miniopterus gleni* | MR220 | Madagascar |
| **Hsp23** | **MW039229** | *Miniopterus griveaudi* | MR69 | Madagascar |
| **Hsp24** | **MW039230** | *Miniopterus ambohitrensis* | MR194 | Madagascar |
| 47MG | MH744532 | *Myotis goudoti* | FMNH 217967 | Madagascar |
| 493MG | MH744533 | *Myotis goudoti* | FMNH 221321 | Madagascar |
| 594MG | MH744534 | *Myotis goudoti* | UABBA 33001 | Madagascar |
| 722MG | MH744535 | *Myotis goudoti* | FMNH 218006 | Madagascar |
| 554MG | MH744536 | *Myotis goudoti* | UADBA 32995 | Madagascar |
|  | HM055583 | *Unspecified bat species* |  | Switzerland |
|  | HM055584 | *Unspecified bat species* |  | Switzerland |
|  | HM055585 | *Unspecified bat species* |  | Switzerland |
|  | HM055586 | *Unspecified bat species* |  | Switzerland |
|  | AY762075 | *Myotis goudoti* | FMNH 175810 | Madagascar |
| **Hsp26** | **MW039232** | *Myotis goudoti* | MR161 | Madagascar |
| **Hsp27** | **MW039233** | *Myotis goudoti* | SMG19285 | Madagascar |
|  | KF159699 | *Miniopterus villiersi* |  | Guinea |
|  | KF159681 | *Miniopterus villiersi* |  | Guinea |
|  | KF 159675 | *Miniopterus villiersi* |  | Guinea |
|  | AY762070 | *Miniopterus manavi* |  | Madagascar |
|  | JQ995285 | *Miniopterus inflatus* |  | Gabon |
|  | JQ995284 | *Miniopterus inflatus* |  | Gabon |
|  | JQ995286 | *Miniopterus inflatus* |  | Gabon |
|  | JQ995287 | *Miniopterus inflatus* |  | Gabon |
|  | JQ995288 | *Miniopterus inflatus* |  | Gabon |
|  | JN990708 | *Miniopterus schreibersii* |  | Switzerland |
|  | JN990709 | *Miniopterus schreibersii* |  | Switzerland |
|  | JN990710 | *Miniopterus schreibersii* |  | Switzerland |
|  | JN990711 | *Miniopterus schreibersii* |  | Switzerland |
|  | KF159714 | *Pipistrellus* aff. *grandidieri* |  | Guinea |
|  | KF159700 | *Laephotis capensis* |  | Guinea |
|  | EF179354 | *Kerivoula hardwickii* |  | Cambodia |
| **Hsp25** | **MW039231** | *Scotophilus robustus* | MR35 | Madagascar |
|  | MT750305 | *Scotophilus kuhlii* | CC-28 | Thailand |
|  | MT750306 | *Scotophilus kuhlii* | CC-31 | Thailand |
|  | MT750307 | *Scotophilus kuhlii* | CC-33 | Thailand |
|  | MT750308 | *Scotophilus kuhlii* | CC-41 | Thailand |
|  | MT750309 | *Scotophilus kuhlii* | CC-45 | Thailand |
