# Supplementary material for: Diversity, distribution, and drivers of polychromophilus infection in Malagasy bats
Source: Malar J. 2021 Mar 20;20:157. doi: 10.1186/s12936-021-03696-0 (PMC7980569; doi:10.1186/s12936-021-03696-0)
Supplement: Supplementary file 3 — Additional file 3: Table S3. Localities with positive occurrence of Polychromophilus in Madagascar including the origin of the sample (cave or trapping) and their location (latitude, longitude). [file 12936_2021_3696_MOESM3_ESM.docx]

Table S3. Localities with positive occurrence of *Polychromophilus* in Madagascar including the origin of the sample (cave or trapping) and their location (latitude, longitude).

| **Pays** | **Province** | **locality** | **Origin** | **latitude** | **longitude** | **P1** | **P2** | **P3** |
| --- | --- | --- | --- | --- | --- | --- | --- | --- |
| Madagascar | Toliara | Grotte de Bekoaky, 9.4 km SSE Ankililaoka | cave | -22,773°S | 43,723°E | 1 | 1 | 0 |
| Madagascar | Toliara | Grotte de Tanambao (Bishiko) 0.75 km E St Augustin | cave | -23,549°S | 43,767°E | 1 | 0 | 0 |
| Madagascar | Toliara | grotte d'Ambanilia 3.7 km SSE Sarodrano | cave | -23,540°S | 43,746°E | 1 | 0 | 0 |
| Madagascar | Fianarantsoa | Grotte d'Andranomilitra, west of Ihosy off RN7 | cave | -22,385°S | 46,056°E | 1 | 0 | 0 |
| Madagascar | Fianarantsoa | grotte de Fandanana, 4.1 km NE de Fandriana | cave | -20,183°S | 47,386°E | 1 | 1 | 0 |
| Madagascar | Fianarantsoa | Parc National d'Isalo, 3.8 km NW de Ranohira, along Namaza river | Trapping | -22,540°S | 45,380°E | 1 | 0 | 0 |
| Madagascar | Fianarantsoa | Parc National d'Isalo, Grotte de Bekapity | cave | -22,633°S | 45,218°E | 0 | 0 | 0 |
| Madagascar | Toliara | Parc National de Tsimanampetsotsa, Grotte d'Andranoilovy | cave | -24,050°S | 43,750°E | 1 | 0 | 0 |
| Madagascar | Toliara | grotte de Vintane, (Vintany) 4.1 km SE Itampolo | cave | -24,702°S | 43,964°E | 1 | 0 | 0 |
| Madagascar | Mahajanga | Grotte d'Anjohikely (south entrance), 1.5 km NE Antanamarina | cave | -15,561°S | 46,874°E | 1 | 0 | 0 |
| Madagascar | Mahajanga | Grotte d'Anjohibe-3.7 km NE Antanamarina | cave | -15,538°S | 46,886°E | 1 | 0 | 0 |
| Madagascar | Mahajanga | Grotte d'Anjohikely , 1.6 km NE Antanamarina | cave | -15,559°S | 46,878°E | 1 | 0 | 0 |
| Madagascar | Antsiranana | Parc National d'Ankarana, Ambahibe Cave | cave | -12,968°S | 49,121°E | 1 | 1 | 0 |
| Madagascar | Antsiranana | Parc National d'Ankarana, Grotte d'Andrafiabe | cave | -12,932°S | 49,060°E | 1 | 1 | 0 |
| Madagascar | Antsiranana | Parc National d'Ankarana, in forest near Andrafiabe Cave | Trapping | -12,932°S | 49,057°E | 1 | 1 | 0 |
| Madagascar | Antsiranana | Parc National d'Ankarana, Grotte d'Ambatoharanana | cave | -12,988°S | 49,022°E | 1 | 0 | 0 |
| Madagascar | Antananarivo | Réserve Spéciale d'Ambohitantely | cave | -18,181°S | 47,289°E | 1 | 1 | 0 |
| Madagascar | Antananarivo | Réserve Spéciale d'Ambohitantely | Trapping | -18,181°S | 47,289°E | 0 | 1 | 0 |
| Madagascar | Mahajanga | Parc National de Bemaraha | cave | -19,010°S | 44,768°E | 1 | 1 | 0 |
| Madagascar | Fianarantsoa | At the edge of Parc National of Isalo | Trapping | -22,548°S | 45,399°E | 1 | 1 | 0 |
| Madagascar | Toamasina | Réserve de Betampona | Trapping | -17,557°S | 49,122°E | 0 | 1 | 0 |
| Madagascar | Fianarantsoa | Station Forestière de Kianjavato | Trapping | -21,379°S | 47,869°E | 1 | 0 | 1 |
| Madagascar | Antsiranana | Masoala, Sahabe | Trapping | -15,334°S | 50,165°E | 0 | 1 | 0 |
| Madagascar | Antsiranana | Marojejy, Antsahabe | Trapping | -14,274°S | 49,466°E | 1 | 0 | 0 |
| Madagascar | Antsiranana | Marojejy, Beamalona | Trapping | -14,271°S | 49,462°E | 1 | 0 | 0 |
| Madagascar | Antsiranana | Marojejy, Mandena | Trapping | -14,273°S | 49,481°E | 1 | 0 | 0 |
| Madagascar | Antsiranana | Marojejy_Antanimbaribe | Trapping | -14,294°S | 49,355°E | 1 | 0 | 0 |
